# Urban-rural disparity of overweight/obesity distribution and its potential trend with breast cancer among Chinese women

**DOI:** 10.18632/oncotarget.10968

**Published:** 2016-07-30

**Authors:** Ying Gao, Yubei Huang, Fengju Song, Hongji Dai, Peishan Wang, Haixin Li, Hong Zheng, Henglei Dong, Jiali Han, Yaogang Wang, Kexin Chen

**Affiliations:** ^1^ Department of Health Service Management, School of Public Health, Tianjin Medical University, Tianjin, China; ^2^ Health Management Center, Tianjin Medical University General Hospital, Tianjin, China; ^3^ Department of Epidemiology and Biostatistics, Key Laboratory of Cancer Prevention and Therapy, Tianjin, Key Laboratory of Breast Cancer Prevention and Therapy, Ministry of Education, National Clinical Research Center for Cancer, Tianjin Medical University Cancer Institute and Hospital, Tianjin, China; ^4^ Department of Epidemiology, Fairbanks School of Public Health, Simon Cancer Center, Indiana University, Indianapolis, Indiana, USA

**Keywords:** overweight, obesity, disparity, breast cancer

## Abstract

**Objective:**

To evaluate the urban-rural disparity of overweight/obesity and explore its potential trend with breast cancer among Chinese women.

**Results:**

The prevalence of overweight/obesity for Chinese rural women (35.2%, 29.2% for overweight and 6.0% for obesity) was significantly higher than that for Chinese urban women (33.4%, 27.7% for overweight and 5.7% for obesity) (*P* < 0.001). For either rural or urban women, the prevalence of overweight/obesity was highest in north region, followed by east region for rural women and north-east region for urban women. For rural women, higher prevalence of overweight/obesity was significantly positively associated with elder age, Han nationality, low level of education, no occupation, high family income, less number of family residents, insurance, and elder age at marriage. Similar positive associations were also found for urban women, except negative associations for high family income, less number of family residents, and elder age at marriage. A non-significant positive trend between overweight/obesity and breast cancer was found for rural women [odds ratio (OR): 1.06; 95% confidence interval (CI): 0.87–1.29], but a significant positive trend for urban women (OR: 1.55; 95% CI: 1.19–2.02).

**Materials and Methods:**

A total of 1 210 762 participants were recruited from the Chinese National Breast Cancer Screening Program. Overweight and obesity were defined as body mass index (BMI) ranged 24.0–27.9 kg/m^2^ and BMI ≥ 28.0kg/m^2^, respectively.

**Conclusions:**

There was an obvious urban-rural disparity of overweight/obesity distribution among Chinese women, which could also lead to an obvious disparity of breast cancer distribution.

## INTRODUCTION

Obesity is a known risk factor for various chronic diseases and mortality, including cancer, such as breast, colon and liver [1–3]. The Global Burden of Disease Study estimated that at least 2.1 billion adults were overweight, including 671 million people with obesity in 2013 [4]. In terms of global disability-adjusted life years (DALYs) in 2013, high body mass index (BMI) caused 4.4 million deaths and 134.0 million DALYs, and ranked as the third most important contributor to DALYs worldwide 5. Due to the increasing burden of disease caused by obesity [1–5], obesity has been defined as an independent chronic disease by the World Health Organization (WHO) [6]. As in the west, many Asian countries, for example China, are experiencing a steep rise in the prevalence of overweight/obesity and obesity associated diseases in their populations [7–10].

Before 1990s, the etiologic studies of obesity mainly focused on dietary and behavioral factors [11, 12], few of them paid attention to the socio-demographic factors. Since Sobal and his colleague first systematically reviewed the relationship between socio-demographic factors and obesity [13], this topic had quickly become a hot spot in the field of obesity research. And several studies had found an inverse relationship between obesity and lower socioeconomic status, including low education, low income, no occupation, and so on [14–16]. In addition, the relationship had shown a potential region disparity, which indicated that different risk factors may be responsible for the different prevalence of obesity for different regions. However, most studies were conducted in developed countries [17–20], few of them were conducted in developing countries [21–23].

China, as the world's most populous country and the largest developing country, is also experiencing rapid economic, social, and cultural development, especially in urban areas. These rapid developments not only result in dramatic changes in lifestyles and dietary pattern, but also a greatly increased burden of obesity and other chronic diseases associated with these rapid changes [24, 25]. As the urban-rural disparity in socioeconomic level is enlarging, the urban-rural disparities in overweight/obesity and obesity-associated diseases are also increasing rapidly [26]. However, few studies focus on these urban-rural disparities in overweight/obesity and obesity-associated diseases, and explore the potential risk factors of these disparities, especially in the largest developing country.

Therefore, in the present study, we aim to examine the urban-rural disparity of overweight/obesity distribution and its potential trend with breast cancer among Chinese women, based on a large population-based survey of women from the Chinese National Breast Cancer Screening Program.

## RESULTS

### Demographic characteristics of study participants

A total of 1 210 762 participants were included in the final analyses. Among these participants, 397 419 were urban residents and 813 343 were rural residents. Among Chinese urban women, the proportions of Han nationality (96.6%), a high level of education (20.5%), having an occupation (94.8%), high family income (26.7%), less number of family residents (67.7%), having insurance (80.4%), and marriage at an older age (27.6%) were significantly higher than those among Chinese rural women (the corresponding proportions were 87.1%, 2.7%, 20.9%, 13.7%, 37.4%, 96.9%, and 7.9%, respectively).

### Prevalence of overweight/obesity

According to Chinese criteria, the prevalence of overweight/obesity for Chinese rural women (35.2%, 29.2% for overweight and 6.0% for obesity) was significantly higher than that for Chinese urban women (33.4%, 27.7% for overweight and 5.7% for obesity) (*P* < 0.001; Table 1). According to WHO criteria, the corresponding prevalence of overweight/obesity for rural women (23.1%) was still higher than that for urban women (21.9%) (*P* < 0.001; Table 1).

**Table 1:** Prevalence of overweight/obesity among Chinese women in urban and rural areas according to Chinese criteria and WHO criteria

BMI	Urban			Rural			*P* value
	*N*	Crude proportion, % (95%CI)	Age- standardized proportion (%)^a^	*N*	Crude proportion, % (95%CI)	Age- standardized proportion (%)^a^	
Chinese criteria							
Underweight (BMI <18.5)	18 390	4.6 (4.6–4.7)	4.6	30 648	3.8 (3.7–3.8)	3.8	
Normal (BMI ≥ 18.5 & < 24.0)	246 299	62.0 (61.8–62.1)	61.0	496 570	61.1 (60.9–61.2)	61.2	< 0.001
Overweight (BMI ≥ 24.0 & < 28.0)	110 083	27.7 (27.6–27.8)	28.4	237 160	29.2(29.0*–*29.3)	29.0	
Obesity (BMI ≥ 28.0)	22 647	5.7 (5.6–5.8)	6.0	48 965	6.0 (6.0–6.1)	6.0	
Overweight/Obesity (BMI ≥ 24.0)	132 730	33.4 (33.3–33.5)	34.4	286 125	35.2 (35.1–35.3)	35.0	< 0.001
WHO criteria							
Underweight (BMI <18.5)	18 390	4.6 (4.6–4.7)	4.6	30 648	3.8 (3.7–3.8)	3.8	
Normal (BMI ≥ 18.5 & < 25.0)	292 005	73.5 (73.3–73.6)	72.7	594 630	73.1 (73.0–73.2)	73.2	< 0.001
Overweight (BMI ≥ 25.0 & < 30.0)	78 946	19.9 (19.7–20.0)	20.6	170 043	20.9 (20.8–21.0)	20.8	
Obesity (BMI ≥ 30.0)	8078	2.0 (2.0–2.1)	2.1	18 022	2.2 (2.2–2.2)	2.2	
Overweight/Obesity (BMI ≥ 25.0)	87 024	21.9 (21.8–22.0)	22.7	188 065	23.1 (23.0–23.2)	23.0	< 0.001

a Age-standardized to the 2000 Chinese population.

*P* value, chi-square test.

**Figure 1:**
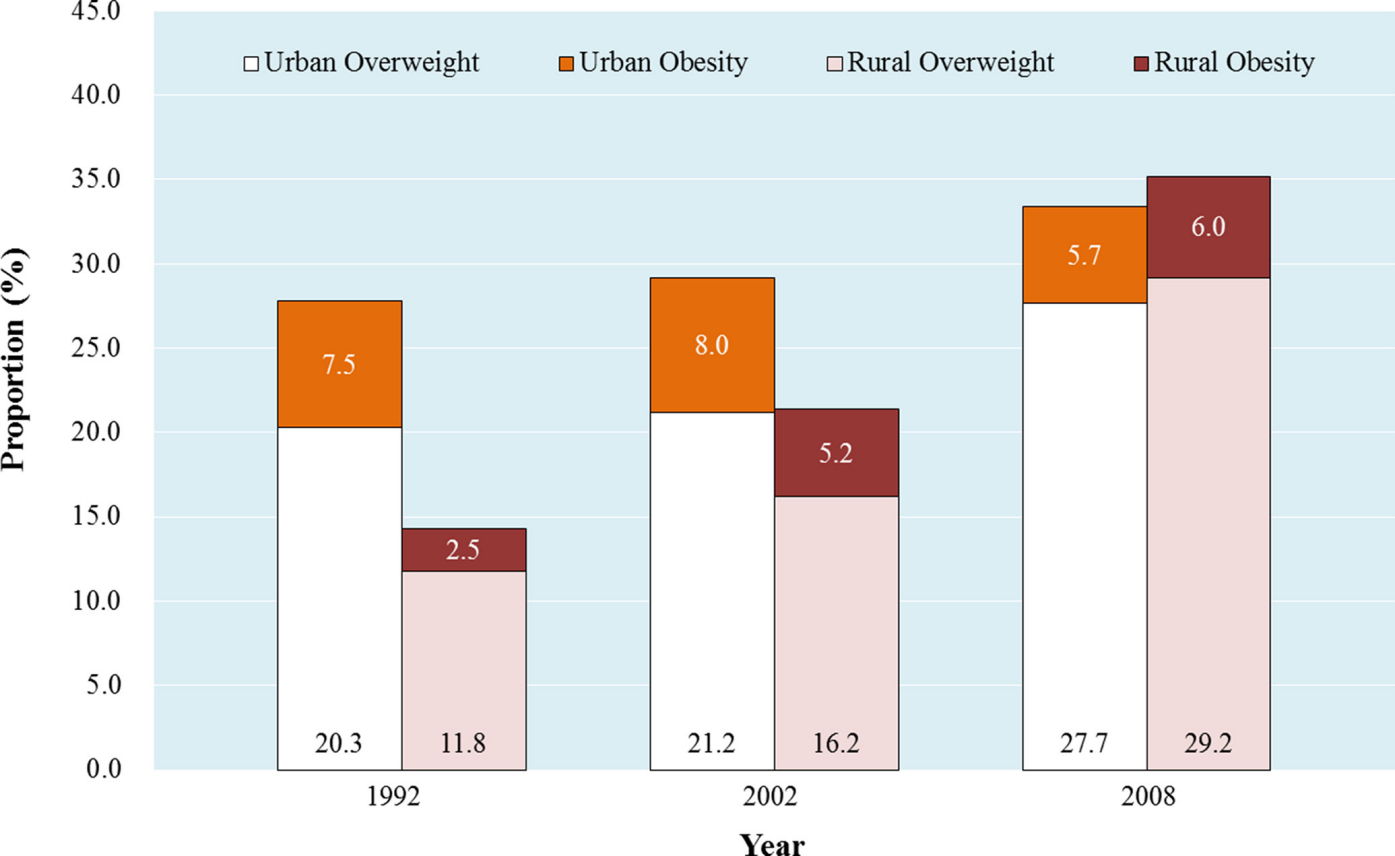
The women prevalence of overweight/obesity from Chinese National Nutrition Survey 1992 (CNNS 1992), Chinese National Nutritional and Health Survey 2002 (CNNHS 2002), and Chinese National Breast Cancer Screening Program 2008 (CNBCSP 2008)

Due to the different sampling frame, we cannot directly compare the prevalence in this study with other Chinese nationally representative data published previously. However, a crude increasing trend for the prevalence of overweight/obesity could be observed across the data. Compared with the prevalence of overweight/obesity from Chinese National Nutrition Survey 1992 (CNNS 1992), the prevalence of overweight/obesity crudely increased 5.6% for urban women (from 27.8% in 1992 to 33.4% in 2008) and 20.9% for rural women (from 14.3% in 1992 to 35.2% in 2008), respectively (Figure 1). In both urban and rural women in 2008, the ratios of overweight and obesity were approximately to 5.0, which indicated a potentially huge obesity population would emerge from the overweight population either in Chinese urban or rural regions unless an urgent strategy is undertaken.

**Figure 2:**
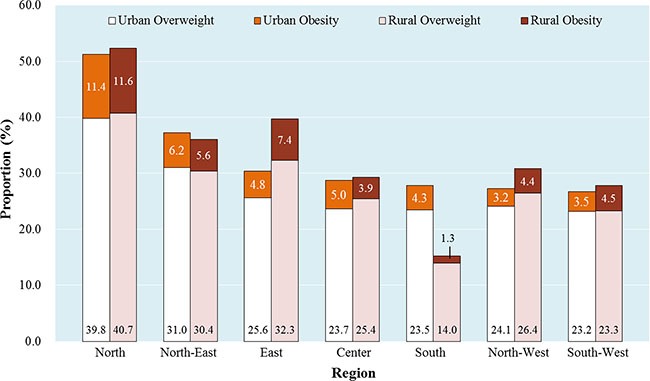
Regional prevalence of overweight/obesity among Chinese urban and rural women Note: North included Beijing, Tianjin, Hebei, Inner Mongolia, Shanxi. North-East included Liaoning, Heilongjiang, Jilin. East included Shandong, Anhui, Jiangsu, Zhejiang, Shanghai, Fujian. Center included Jiangxi, Henan, Hubei, Hunan. South included Guangxi, Hainan. North-West included Gansu, Ningxia, Qinghai, Shaanxi, Xinjiang. South-West included Guizhou, Sichuan, Chongqing, Yunnan.

**Figure 3:**
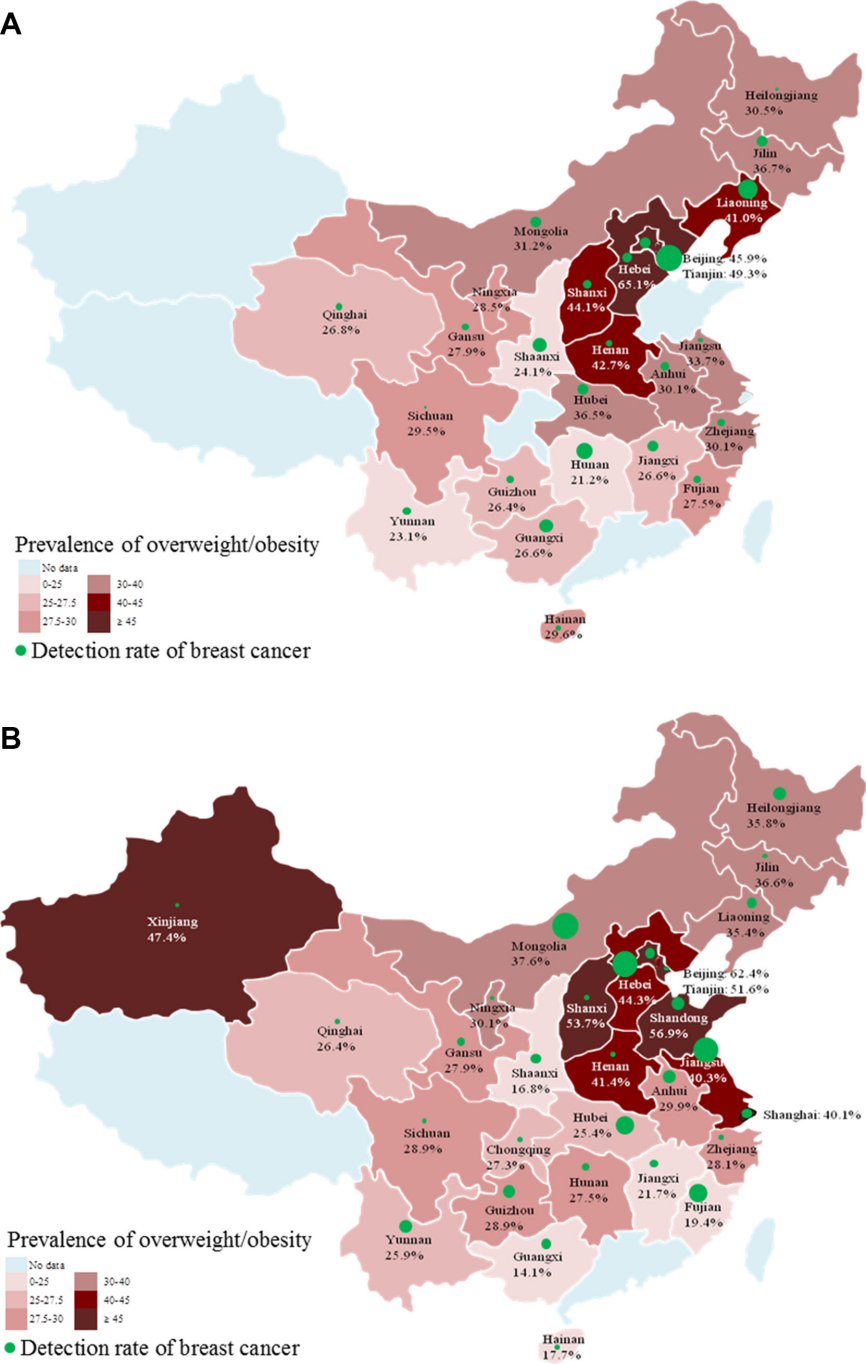
The provincial prevalence of overweight/obesity and the corresponding detection rate of breast cancer (1/10^5^) among Chinese urban (A) and rural (B) women Note: The size of green bubbles corresponded with the size of provincial detection rate of breast cancer.

For either rural or urban women, the prevalence of overweight/obesity was highest in the northern region (52.3% vs. 51.2%), followed by the eastern region for rural women (39.7%) and north-eastern regions for urban women (37.2%), respectively (Figure 2). According to the provincial distribution of overweight/obesity shown in Figure 3, the urban areas in Hebei, Tianjin, Beijing, Shanxi, Henan, and Liaoning showed obviously higher prevalence of overweight/obesity than urban areas in other provinces (Figure 3A). And the rural areas in Beijing, Shandong, Shanxi, Tianjin, Hebei, Henan, Jiangsu, and Xinjiang showed obviously higher prevalence of overweight/obesity than rural areas in other provinces (Figure 3B).

### Association between overweight/obesity and demographic characteristics

As shown in Table 2 and Table 3, the prevalence of overweight/obesity showed a significantly increasing trend with age both for urban and rural women (both *P*_trend_ < 0.001). In rural areas, higher proportions of overweight/obesity were more likely to be positively associated with Han nationality [vs. others nationality, odds ratio (OR): 1.18; 95% confidence interval (CI): 1.16–1.20], high school education (vs. college or above, OR: 1.23; 95% CI: 1.20–1.27) and primary school education or below (vs. college or above, OR: 1.25; 95% CI: 1.22–1.29), no occupation (vs. with occupation, OR: 1.02; 95% CI: 1.01–1.03), family income of 1000– 3000 RMB/month (vs. 1000 RMB/month or below, OR: 1.09; 95% CI: 1.07–1.10) and 3000 RMB/month or above (vs. 1000 RMB/month or below, OR: 1.16; 95% CI: 1.15–1.18), 1–3 family residents (vs. 4 family residents or above, OR: 1.04; 95% CI: 1.03–1.05), with insurances (vs. no insurance, OR: 1.13; 95% CI: 1.10–1.17), and the age at marriage being more than 26 years old (vs. 20 years or below, OR: 1.17; 95% CI: 1.15–1.20) and 21–25 years old (vs. 20 years or below, OR: 1.10; 95% CI: 1.09–1.11).

**Table 2:** Prevalence of overweight/obesity among Chinese rural women according to different demographic characteristics

Variable	BMI category (*N*, %)				*P*_1_	*P*_trend_	*P*_2_	OR (95%CI)^a^
	Underweight (BMI < 18.5)	Normal (BMI ≥ 18.5 & < 24.0)	Overweight (BMI ≥ 24.0 & < 28.0)	Obesity (BMI ≥ 28.0)				
Age (years)								
35–39	10 406 (5.1)	139 496 (68.1)	46 762 (22.8)	8076 (3.9)	Ref.		Ref.	1.00
40–44	7033 (3.5)	125 940 (62.4)	58 121 (28.8)	10 808 (5.4)	< 0.001		< 0.001	1.42 (1.40–1.44)
45–49	5593 (3.0)	108 517 (58.4)	59 215 (31.8)	12 607 (6.8)	< 0.001		< 0.001	1.72 (1.70–1.74)
50–54	3511 (3.2)	61 526 (55.3)	37 378 (33.6)	8830 (7.9)	< 0.001		< 0.001	1.94 (1.91–1.97)
55–59	4105 (3.7)	61 091 (55.8)	35 684 (32.6)	8644 (7.9)	< 0.001	< 0.001	< 0.001	1.86 (1.83–1.89)
Nationality								
Others	4446 (5.4)	51 925 (62.8)	21 648 (26.2)	4640 (5.6)	Ref.		Ref.	1.00
Han	25 696 (3.6)	431 298 (60.9)	208 436 (29.4)	42 984 (6.1)	< 0.001	—	< 0.001	1.18 (1.16–1.20)
Unknown	506 (2.3)	13 347 (59.9)	7076 (31.8)	1341 (6.0)				
Education								
≥ College	910 (4.1)	14 321 (65.3)	5708 (26.0)	994 (4.5)	Ref.		Ref.	1.00
High school	14 208 (3.5)	246 659 (61.3)	117 282 (29.2)	24 021 (6.0)	< 0.001		< 0.001	1.23 (1.20–1.27)
≤ Primary school	14 954 (4.0)	223 367 (60.5)	108 298 (29.3)	22 879 (6.2)	< 0.001	< 0.001	< 0.001	1.25 (1.22–1.29)
Unknown	576 (2.9)	12 223 (61.9)	5872 (29.7)	1071 (5.4)				
Occupation								
Yes	5840 (3.4)	104 714 (61.6)	49 256 (29.0)	10 312 (6.1)	Ref.		Ref.	1.00
No	24 543 (3.9)	387 064 (60.8)	186 370 (29.3)	38 399 (6.0)	< 0.001	—	0.02	1.02 (1.01–1.03)
Unknown	265 (3.9)	4792 (70.0)	1534 (22.4)	254 (3.7)				
Family income (RMB/month)^b^								
< 1000	10 674 (4.4)	148 122 (61.6)	67 598 (28.1)	13 971 (5.8)	Ref.		Ref.	1.00
1000–3000	15 253 (3.4)	271 187 (60.8)	132 595 (29.7)	27 085 (6.1)	< 0.001		< 0.001	1.09 (1.07–1.10)
≥ 3000	3432 (3.1)	66 213 (59.5)	34 192 (30.7)	7443 (6.7)	< 0.001	< 0.001	< 0.001	1.16 (1.15–1.18)
Unknown	1289 (8.3)	11 048 (70.9)	2775 (17.8)	466 (3.0)				
Family resident (persons)								
≥ 4	19 144 (3.8)	304 813 (61.2)	145 239 (29.2)	28 943 (5.8)	Ref.		Ref.	1.00
1–3	11 111 (3.6)	184 361 (60.5)	89 427 (29.4)	19 632 (6.4)	< 0.001	—	< 0.001	1.04 (1.03–1.05)
Unknown	393 (3.7)	7396 (69.3)	2494 (23.4)	390 (3.7)				
Insurance								
No	635 (3.4)	11 905 (64.0)	5049 (27.2)	999 (5.4)	Ref.		Ref.	1.00
Yes	29 801 (3.8)	480 049 (60.9)	230 573 (29.3)	47 663 (6.0)	< 0.001	< 0.001	< 0.001	1.13 (1.10–1.17)
Unknown	212 (3.2)	4616 (69.2)	1538 (23.1)	303 (4.5)				
Marriage								
Married	29 424 (3.7)	485 010 (61.0)	232 806 (29.3)	48 002 (6.0)	Ref.		Ref.	1.0
Others^c^	477 (4.9)	5752 (59.0)	2826 (29.0)	698 (7.2)	< 0.001	—	0.09	1.04 (0.99–1.08)
Unknown	747 (8.9)	5808 (69.6)	1528 (18.3)	265 (3.2)				
Age at marriage (years)								
≤ 20	8160 (4.3)	118 448 (62.2)	53 170 (27.9)	10 739 (5.6)	Ref.		Ref.	1.00
21–25	19 521 (3.5)	334 697 (60.8)	163 282 (29.7)	33 098 (6.0)	< 0.001		< 0.001	1.10 (1.09–1.11)
≥ 26	2544 (4.0)	37 688 (58.8)	18 964 (29.6)	4851 (7.6)	< 0.001	< 0.001	< 0.001	1.17 (1.15–1.20)
Unknown	423 (5.2)	5737 (70.1)	1744 (21.3)	277 (3.4)				

BMI, body mass index.

*P*_1_, *P* values of chi-square test for BMI distribution with four categories (Less weight, Normal, Overweight, Obesity).

*P*_2_, *P* values of univariate logistic regression for BMI distribution with two categories (Less weight/Normal, and Overweight/Obesity).

a ORs of Overweight/Obesity for the specific subgroups compared with the reference subgroup.

b According to the exchange rate of Chinese RMB to U.S. dollar in 2008 (1 RMB = 0.1611 USD), 1000 RMB = 161.1 USD, 3000 RMB = 483.3 USD.

c Others included unmarried, separated, divorced and widowed.

**Table 3:** Prevalence of overweight/obesity among Chinese urban women according to different demographic characteristics

Variable	BMI category (*N*, %)				*P*_1_	*P*_trend_	*P*_2_	OR (95%CI)^a^
	Underweight (BMI < 18.5)	Normal (BMI ≥ 18.5 & < 24.0)	Overweight (BMI ≥ 24.0 & < 28.0)	Obesity (BMI ≥ 28.0)				
Age (years)								
35–39	7023 (7.3)	69 021 (71.4)	17 608 (18.2)	2956 (3.1)	Ref.		Ref.	1.00
40–44	3975 (4.5)	59 002 (66.8)	21 509 (24.4)	3802 (4.3)	< 0.001		< 0.001	1.49 (1.46–1.52)
45–49	2609 (3.5)	44 761 (60.3)	22 374 (30.1)	4510 (6.1)	< 0.001		< 0.001	2.10 (2.05–2.14)
50–54	1875 (3.2)	32 354 (55.8)	19 581 (33.8)	4204 (7.2)	< 0.001		< 0.001	2.57 (2.51–2.63)
55–59	1358 (3.2)	21 741 (51.8)	15 218 (36.3)	3625 (8.6)	< 0.001		< 0.001	3.02 (2.94–3.09)
60–64	908 (3.8)	12 025 (50.0)	8835 (36.8)	2259 (9.4)	< 0.001		< 0.001	3.17 (3.08–3.27)
65–69	642 (4.5)	7395 (51.8)	4958 (34.7)	1291 (9.0)	< 0.001	< 0.001	< 0.001	2.88 (2.77–2.98)
Nationality								
Others	570 (4.8)	7544 (64.1)	3076 (26.1)	575 (4.9)	Ref.		Ref.	1.00
Han	17 755 (4.6)	237 537 (61.9)	106 521 (27.8)	21 977 (5.7)	< 0.001	—	< 0.001	1.12 (1.07–1.16)
Unknown	65 (3.5)	1218 (65.3)	486 (26.1)	95 (5.1)				
Education								
≥ College	4511 (5.5)	57 946 (71.3)	16 577 (20.4)	2292 (2.8)	Ref.		Ref.	1.00
High school	10 522 (4.5)	144 443 (61.8)	65 568 (28.0)	13 380 (5.7)	< 0.001		< 0.001	1.69 (1.66–1.72)
≤ Primary school	3312 (4.1)	43 391 (53.3)	27 693 (34.0)	6937 (8.5)	< 0.001	< 0.001	< 0.001	2.45 (2.40–2.51)
Unknown	45 (5.3)	519 (61.3)	245 (28.9)	38 (4.5)				
Occupation								
Yes	17 694 (4.7)	236 749 (62.9)	102 311 (27.2)	19 862 (5.3)	Ref.		Ref.	1.00
No	659 (3.3)	9100 (45.4)	7544 (37.6)	2742 (13.7)	< 0.001	—	< 0.001	2.20 (2.13–2.26)
Unknown	37 (4.9)	450 (59.4)	228 (30.1)	43 (5.7)				
Family income (RMB/month)^b^								
< 1000	3783 (5.0)	45 669 (59.9)	21 827 (28.6)	5013 (6.6)	Ref.		Ref.	1.00
1000–3000	9592 (4.5)	130 896 (61.6)	59 830 (28.1)	12 330 (5.8)	< 0.001		< 0.001	0.95 (0.93–0.96)
≥ 3000	4916 (4.6)	68 215 (64.3)	27 739 (26.2)	5143 (4.9)	< 0.001	< 0.001	< 0.001	0.83 (0.81–0.85)
Unknown	99 (4.0)	1519 (61.6)	687 (27.9)	161 (6.5)				
Family resident (persons)								
≥ 4	4810 (4.5)	59 769 (56.3)	33 532 (31.6)	7961 (7.5)	Ref.		Ref.	1.00
1–3	12 593 (4.7)	172 258 (64.0)	70 770 (26.3)	13 481 (5.0)	< 0.001	—	< 0.001	0.71 (0.70–0.72)
Unknown	987 (4.4)	14 272 (64.2)	5781 (26.0)	1205 (5.4)				
Insurance								
No	3281 (5.9)	34 908 (62.8)	14 514 (26.1)	2859 (5.1)	Ref.		Ref.	1.00
Yes	14 123 (4.4)	197 267 (61.7)	89756 (28.1)	18557 (5.8)	< 0.001	—	< 0.001	1.13 (1.11–1.15)
Unknown	986 (4.5)	14 124 (63.8)	5813 (26.2)	1231 (5.6)				
Marriage								
Married	17 408 (4.6)	236 102 (62.0)	105 663 (27.7)	21 625 (5.7)	Ref.		Ref.	1.00
Others^c^	966 (6.0)	9920 (61.2)	4316 (26.6)	1009 (6.2)	< 0.001	—	0.13	0.97 (0.94–1.01)
Unknown	16 (3.9)	277 (67.6)	104 (25.4)	13 (3.2)				
Age at marriage (years)								
≤ 20	1472 (4.1)	19 944 (56.1)	11 364 (31.9)	2789 (7.8)	Ref.		Ref.	1.00
21–25	10 373 (4.3)	151 666 (62.2)	68 025 (27.9)	13 765 (5.6)	< 0.001		< 0.001	0.76 (0.75–0.78)
≥ 26	6059 (5.5)	69 455 (63.3)	28 616 (26.1)	5622 (5.1)	< 0.001	< 0.001	< 0.001	0.69 (0.67–0.70)
Unknown	486 (5.9)	5234 (63.3)	2078 (25.1)	471 (5.7)				

BMI, body mass index.

*P*_1_, *P* values of chi-square test for BMI distribution with four categories (Less weight, Normal, Overweight, Obesity).

*P*_2_, *P* values of univariate logistic regression for BMI distribution with two categories (Less weight/Normal, and Overweight/Obesity).

a ORs of Overweight/Obesity for the specific subgroups compared with the reference subgroup.

b According to the exchange rate of Chinese RMB to U.S. dollar in 2008 (1 RMB = 0.1611 USD), 1000 RMB = 161.1 USD, 3000 RMB = 483.3 USD.

c Others included unmarried, separated, divorced and widowed.

Similar positive associations were also found among Chinese urban women, except negative association for family income of 1000–3000 RMB/month (vs. 1000 RMB/month or below, OR: 0.95; 95% CI: 0.93–0.96) and 3000 RMB/month or above (vs. 1000 RMB/month or below, OR: 0.83; 95% CI: 0.81–0.85), 1–3 family residents (vs. 4 family residents or above, OR: 0.71; 95% CI: 0.70–0.72), and the age at marriage being more than 26 years old (vs. 20 years or below, OR: 0.69; 95% CI: 0.67–0.70) and 21–25 years old (vs. 20 years or below, OR: 0.76; 95% CI: 0.75–0.78).

### Trend between overweight/obesity and breast cancer

A non-significant positive trend between overweight/obesity and breast cancer was found for rural women (OR: 1.06; 95% CI: 0.87–1.29), but a significant positive trend for urban women (OR: 1.55; 95% CI: 1.19–2.02) (Table 4, Figure 3). After stratifying by socio-demographic variables, the positive trend was still observed between overweight/obesity and breast cancer in urban women, but not statistically significant in rural women (Supplementary Table S1).

**Table 4:** Trends between overweight/obesity and risk of breast cancer among Chinese urban and rural women

BMI	Breast cancer			*P*_1_	*P*_2_	OR (95%CI)
	Case	Control	Detected rate (1/10^5^)			
Urban						
Normal (BMI ≥ 18.5 & < 24.0)	117	246 182	47.5		Ref.	1.00
Underweight (BMI < 18.5)	8	18 382	43.5	0.004	0.81	0.92 (0.45–1.88)
Overweight (BMI ≥ 24.0 & < 28.0)	75	110 008	68.1		0.02	1.44 (1.10–1.92)
Obesity (BMI ≥ 28.0)	22	22 625	97.1		0.002	2.05 (1.30–3.23)
Less weight/Normal	125	264 564	47.2	0.001	Ref.	1.00
Overweight/Obesity	97	132 633	73.1		0.001	1.55 (1.19–2.02)
Rural						
Normal (BMI ≥ 18.5 & < 24.0)	256	496 314	51.6		Ref.	1.00
Underweight (BMI < 18.5)	17	30 631	55.5	0.77	0.77	1.08 (0.66–1.76)
Overweight (BMI ≥ 24.0 & < 28.0)	134	237 026	56.5		0.39	1.10 (0.89–1.35)
Obesity (BMI ≥ 28.0)	23	48 942	47.0		0.67	0.91 (0.60–1.40)
Less weight/Normal	273	526 945	51.8	0.56	Ref.	1.00
Overweight/Obesity	157	285 968	54.9		0.56	1.06 (0.87–1.29)

BMI, body mass index.

*P*_1_, *P* value of chi-square test.

*P*_2_, *P* value of univariate logistic regression.

## DISCUSSION

In this study, we examined the urban-rural disparity of overweight/obesity distribution and its potential trend with breast cancer among Chinese women. Our findings suggested that the prevalence of overweight/obesity for Chinese rural women was significantly higher than that for Chinese urban women. This urban-rural disparity of overweight/obesity distribution could not only be found in the different regions, but also be found according to different demographic factors. Moreover, this urban-rural disparity of overweight/obesity distribution could also lead to a disparity of breast cancer distribution.

Several previous studies had also reported the disparity of overweight/obesity distribution. But most studies focused on the sex disparity or race/ethnic disparity in overweight/obesity distribution [14, 27], few studies focused on the urban-rural disparity. Several reasons could be responsible for this urban-rural disparity of overweight/obesity distribution. The most important reason could be the different social economic level between the urban areas and rural areas in China. As reported in the previous results, the urban women were more likely to have a high level of education, an occupation, high family income, few family residents and marriage at an older age when compared with rural women. These different urban-rural social economic levels could lead to different urban-rural lifestyle and dietary pattern. As pointed out in the previous studies, the dietary pattern shifted away from high-carbohydrate diets toward high-fat, high energy-density foods as income improved, particularly in the low- and middle-income group [28–30]. This phenomenon was worse in Chinese rural women. As reported from the Chinese National Nutritional Survey in 1982 and in 2002, the daily dietary fat intake increased 33g for Chinese rural women (17g for urban women) during the 20 years, and the increments of daily dietary meat and animal oil were 46 g and 0.9g (42 g and decrement of 0.2g for urban women), respectively [31]. Moreover, the available evidences also suggested that higher income indicated more chance of accessing the healthier diet [16, 32]. However, for Chinese rural population, although the increasing family income allows them to access more food, they spend more money on high-fat and high-energy foods rather than on a healthier diet [6]. That's why there was a negative association between high family income and overweight/obesity for urban women, but a positive association for rural women. In addition, this urban-rural disparity of overweight/obesity may also be a result of differences in knowledge and lifestyles between Chinese urban and rural women. Chinese rural women were more likely to live with their descendants and spend more time on taking care of their descendants when compared with their urban counterparts. Furthermore, in Chinese culture, there is a widespread belief that obesity represents health and prosperity [8]. This belief is more popular among rural women than urban women. Therefore, rural women paid less attention to their weight gain than their urban counterparts.

Another important finding was that potential urban-rural disparity of breast cancer could be the result of urban-rural disparity of overweight/obesity distribution. Although obesity was a known risk factor for breast cancer, the pattern of urban-rural disparity in overweight/obesity distribution (high prevalence for rural women and low prevalence for urban women) was not very similar with the pattern of urban-rural disparity in breast cancer (positive trend between breast cancer and overweight/obesity for urban women but a non-significant positive trend for rural women). These results implicated that future strategies focused on controlling overweight/obesity could also prevent the breast cancer for Chinese urban women, but other strategies besides controlling overweight/obesity should also be emphasized to prevent breast cancer.

This study has several strengths. Firstly, the survey was population-based and the sample size was enough, which allowed a relatively stable estimation for each analysis and stratified analysis according to different demographic variables. Secondly, various training courses and seminars were conducted to ensure standardization of data collection. However, there were also several limitations in this study. Firstly, due to lack of information in dietary habits, sedentary behavior, and physical activity for urban and rural women, we cannot further explore the association between overweight/obesity risk and this information. Secondly, as described in the section of results, due to different sampling frame between this study and previously national representative surveys, results cannot be directly compare with previously national representative data in China. However, the data across these surveys could also remind us the crude increasing trend of overweight/obesity prevalence.

## MATERIALS AND METHODS

### Study population

The Chinese Ministry of Health launched the Chinese National Breast Cancer Screening Program (CNBCSP) which covered 29 provinces/autonomous regions/municipalities since 2008. The program recruited 397 thousand urban women aged 35–69 years and 813 thousand rural women aged 35–59 years. The target population was clustered and selected from two or four communities (one or two communities for either urban areas or rural areas) with high incidence of breast cancer in each province. The targeted communities were chosen by the provincial cancer registry according to the local cancer registry data. The study was approved by Institutional Review Board of Tianjin Medical University Cancer Institute and Hospital, and all participants provided written informed consent.

### Exclusion criteria

In this trial, asymptomatic women who had lived in their residential communities for ≥ 3 years. The pregnant women and lactating less than 6 months women were excluded. Moreover, women who had previously been diagnosed with breast cancer were also excluded.

### Data collection

During the face-to-face interview, demographic data was collected from all the participants with a structured questionnaire. The information included the participants' age, nationality, region, education, occupation, family income, number of family residents, health insurance, marriage status, and age at marriage.

Height was measured to the nearest 0.1 cm without shoes using a portable stadiometer and weight was measured to the nearest 0.1 kg with lightweight clothing on a calibrated beam scale. BMI was calculated as weight in kilograms divided by the square of height in meters (Kg/m^2^). Height and weight were measured by local trained investigators. In the final analyses, subjects who had a BMI either less than 14.0 or more than 50.0 were excluded [5].

### BMI categories

According to the Chinese Working Group on Obesity, BMI < 18.5 kg/m^2^ was considered underweight, BMI ranged from 18.5 kg/m^2^ to 23.9 kg/m^2^ was normal, 24.0–27.9 kg/m^2^ was overweight and 28.0 kg/m^2^ or greater was obesity [33]. In order to provide a comparable data with other regions around the world, the definitions according to WHO suggestions (underweight: BMI < 18.5 kg/m^2^; normal: BMI ≥ 18.5 kg/m^2^ & < 25.0 kg/m^2^; overweight: BMI ≥ 25.0 kg/m^2^ & < 30.0 kg/m^2^; obesity: BMI ≥ 30.0 kg/m^2^) were also used [34].

### Quality control

Various training courses and seminars according to the study protocol were conducted with a train-the-trainer model [35]. Firstly, all provincial investigators who participated in the study were trained to learn the standardized epidemiological interview. Secondly, the local investigators who participated in the study were trained by the provincial investigators. Regular intensive training was also provided for local investigators to ensure adherence to protocols.

All the examination data was double-entered and checked for its consistency. Further comprehensive logic checking was conducted to remove the logic errors between variables before analysis.

### Statistical analysis

Chi-square tests were used to compare the overweight/obesity distribution between different demographic characteristics for urban and rural women. Chi-squared test for trend was used to test whether there was a linear trend between overweight/obesity prevalence and ordinal categorical variable. Odds ratios and 95% CI calculated with univariate logistic regression were used to evaluate the association between demographic characteristics and risk of overweight/obesity, and the trend between overweight/obesity and the risk of breast cancer. All analyses were conducted with SPSS version 19.0. *P* value < 0.05 was considered to be statistically significant.

## CONCLUSIONS

In summary, this study demonstrated an obvious urban-rural disparity in the prevalence of overweight/obesity. And this urban-rural disparity in overweight/obesity distribution could also lead to a potential disparity in breast cancer distribution. More worrisomely, compared with previously national representative data in China, the prevalence of overweight/obesity could increase in the future China. This trend could lead to an increasing burden of obesity and obesity associated diseases, such as breast cancer. Therefore, in the future, different strategies targeting different influential factors of overweight/obesity are needed for Chinese urban and rural women so as to control the increasing burden of obesity and obesity associated diseases.

## SUPPLEMENTARY MATERIALS TABLE




