# Supplementary material for: Urban-rural disparity of overweight/obesity distribution and its potential trend with breast cancer among Chinese women
Source: Oncotarget. 2016 Jul 30;7(35):56608–18. doi: 10.18632/oncotarget.10968 (PMC5302938; doi:10.18632/oncotarget.10968)
Supplement: Supplementary file 1 [file oncotarget-07-56608-s001.pdf]

Urban-rural disparity of overweight/obesity distribution and its potential trend with breast cancer among Chinese women

Supplementary Materials

Supplementary Table S1: Trends between overweight/obesity and risk of breast cancer among Chinese urban and rural women according different demographic characteristics. See Supplementary_Table_S1
