# Supplementary material for: Urban-rural disparity of overweight/obesity distribution and its potential trend with breast cancer among Chinese women
Source: Oncotarget. 2016 Jul 30;7(35):56608–18. doi: 10.18632/oncotarget.10968 (PMC5302938; doi:10.18632/oncotarget.10968)
Supplement: Supplementary file 2 [file oncotarget-07-56608-s002.docx]

**Supplement Table 1: Trends between overweight/obesity and risk of breast cancer among Chinese urban and rural women according different demographic characteristics**

| Variable | Subtype | Urban | | | | |  | Rural | | | | |
| --- | --- | --- | --- | --- | --- | --- | --- | --- | --- | --- | --- | --- |
|  |  | Breast Cancer | | | P value | OR (95%CI) |  | Breast Cancer | | | P value | OR (95%CI) |
|  |  | Case | Control | Detected rate (1/10^5^) |  |  |  | Case | Control | Detected rate (1/10^5^) |  |  |
| Age (years) | < 50 |  |  |  |  |  |  |  |  |  |  |  |
|  | BMI < 24.0 | 68 | 186 323 | 36.5 | 0.30 | 1.00 |  | 169 | 396 816 | 42.6 | 0.07 | 1.00 |
|  | BMI ≥ 24.0 | 33 | 72 726 | 45.4 |  | 1.24 (0.82-1.89) |  | 104 | 195 485 | 53.2 |  | 1.25 (0.98-1.60) |
|  | ≥ 50 |  |  |  |  |  |  |  |  |  |  |  |
|  | BMI < 24.0 | 57 | 78 241 | 72.8 | 0.04 | 1.00 |  | 104 | 130 129 | 79.9 | 0.07 | 1.00 |
|  | BMI ≥ 24.0 | 64 | 59 907 | 106.7 |  | 1.47 (1.03-2.10) |  | 53 | 90 483 | 58.5 |  | 0.73 (0.53-1.02) |
| Nationality | Han |  |  |  |  |  |  |  |  |  |  |  |
|  | BMI < 24.0 | 119 | 255 173 | 46.6 | 0.001 | 1.00 |  | 251 | 456 743 | 54.9 | 0.95 | 1.00 |
|  | BMI ≥ 24.0 | 94 | 128 404 | 73.2 |  | 1.57 (1.20-2.06) |  | 139 | 251 281 | 55.3 |  | 1.01 (0.82-1.24) |
|  | Others |  |  |  |  |  |  |  |  |  |  |  |
|  | BMI < 24.0 | 5 | 8109 | 61.6 | 0.69 | 1.00 |  | 16 | 56 355 | 28.4 | 0.13 | 1.00 |
|  | BMI ≥ 24.0 | 3 | 3648 | 82.2 |  | 1.33 (0.32-5.58) |  | 13 | 26 275 | 49.5 |  | 1.74 (0.84-3.62) |
| Education | ≤ Primary school |  |  |  |  |  |  |  |  |  |  |  |
|  | BMI < 24.0 | 21 | 46 682 | 45.0 | 0.002 | 1.00 |  | 127 | 238 194 | 53.3 | 0.84 | 1.00 |
|  | BMI ≥ 24.0 | 36 | 34 594 | 104.0 |  | 2.31 (1.35-3.96) |  | 72 | 131 105 | 54.9 |  | 1.03 (0.77-1.38) |
|  | High school |  |  |  |  |  |  |  |  |  |  |  |
|  | BMI < 24.0 | 78 | 154 887 | 50.3 | 0.25 | 1.00 |  | 133 | 260 734 | 51.0 | 0.71 | 1.00 |
|  | BMI ≥ 24.0 | 49 | 78 899 | 62.1 |  | 1.23 (0.86-1.76) |  | 76 | 141 227 | 53.8 |  | 1.06 (0.80-1.40) |
|  | ≥ College |  |  |  |  |  |  |  |  |  |  |  |
|  | BMI < 24.0 | 26 | 62 431 | 41.6 | 0.22 | 1.00 |  | 7 | 15 224 | 46.0 | 0.42 | 1.00 |
|  | BMI ≥ 24.0 | 12 | 18 857 | 63.6 |  | 1.53 (0.77-3.03) |  | 5 | 6697 | 74.6 |  | 1.62 (0.52-5.12) |
| Occupation | No |  |  |  |  |  |  |  |  |  |  |  |
|  | BMI < 24.0 | 8 | 9751 | 82.0 | 0.72 | 1.00 |  | 217 | 411 390 | 52.7 | 0.63 | 1.00 |
|  | BMI ≥ 24.0 | 10 | 10 276 | 97.2 |  | 1.19 (0.47-3.01) |  | 125 | 224 644 | 55.6 |  | 1.06 (0.85-1.32) |
|  | Yes |  |  |  |  |  |  |  |  |  |  |  |
|  | BMI < 24.0 | 117 | 254 326 | 46.0 | 0.002 | 1.00 |  | 52 | 110 502 | 47.0 | 0.55 | 1.00 |
|  | BMI ≥ 24.0 | 87 | 122 086 | 71.2 |  | 1.55 (1.17-2.05) |  | 32 | 59 536 | 53.7 |  | 1.14 (0.74-1.78) |
| Family income  (RMB/month) | < 1000 |  |  |  |  |  |  |  |  |  |  |  |
|  | BMI < 24.0 | 23 | 49 429 | 46.5 | 0.17 | 1.00 |  | 64 | 158 732 | 40.3 | 0.57 | 1.00 |
|  | BMI ≥ 24.0 | 19 | 26 821 | 70.8 |  | 1.52 (0.83-2.80) |  | 37 | 81 532 | 45.4 |  | 1.13 (0.75-1.69) |
|  | 1000-3000 |  |  |  |  |  |  |  |  |  |  |  |
|  | BMI < 24.0 | 72 | 140 416 | 51.2 | 0.01 | 1.00 |  | 160 | 286 280 | 55.9 | 0.85 | 1.00 |
|  | BMI ≥ 24.0 | 58 | 72 102 | 80.4 |  | 1.57 (1.11-2.22) |  | 87 | 159 593 | 54.5 |  | 0.98 (0.75-1.27) |
|  | ≥ 3000 |  |  |  |  |  |  |  |  |  |  |  |
|  | BMI < 24.0 | 30 | 73 101 | 41.0 | 0.17 | 1.00 |  | 43 | 69 602 | 61.7 | 0.28 | 1.00 |
|  | BMI ≥ 24.0 | 20 | 32 862 | 60.8 |  | 1.48 (0.84-2.61) |  | 33 | 41 602 | 79.3 |  | 1.28 (0.82-2.02) |
| Family resident  (persons) | 1-3 |  |  |  |  |  |  |  |  |  |  |  |
|  | BMI < 24.0 | 93 | 184 758 | 50.3 | 0.006 | 1.00 |  | 106 | 195 366 | 54.2 | 0.77 | 1.00 |
|  | BMI ≥ 24.0 | 66 | 84 185 | 78.3 |  | 1.56 (1.14-2.14) |  | 62 | 108 997 | 56.8 |  | 1.05 (0.77-1.43) |
|  | ≥ 4 |  |  |  |  |  |  |  |  |  |  |  |
|  | BMI < 24.0 | 29 | 64 550 | 44.9 | 0.09 | 1.00 |  | 161 | 323 796 | 49.7 | 0.47 | 1.00 |
|  | BMI ≥ 24.0 | 29 | 41 464 | 69.9 |  | 1.56 (0.93-2.61) |  | 95 | 174 087 | 54.5 |  | 1.10 (0.85-1.41) |
| Insurance | No |  |  |  |  |  |  |  |  |  |  |  |
|  | BMI < 24.0 | 28 | 38 161 | 73.3 | 0.35 | 1.00 |  | 9 | 12 531 | 71.8 | 0.80 | 1.00 |
|  | BMI ≥ 24.0 | 17 | 17 356 | 97.9 |  | 1.34 (0.73-2.44) |  | 5 | 6043 | 82.7 |  | 1.15 (0.39-3.44) |
|  | Yes |  |  |  |  |  |  |  |  |  |  |  |
|  | BMI < 24.0 | 94 | 211 296 | 44.5 | 0.002 | 1.00 |  | 259 | 509 591 | 50.8 | 0.48 | 1.00 |
|  | BMI ≥ 24.0 | 77 | 108 236 | 71.1 |  | 1.60 (1.18-2.16) |  | 152 | 278 084 | 54.6 |  | 1.08 (0.88-1.31) |
| Age at marriage  (years) | ≤ 20 |  |  |  |  |  |  |  |  |  |  |  |
|  | BMI < 24.0 | 7 | 21 409 | 32.7 | 0.18 | 1.00 |  | 47 | 126 561 | 37.1 | 0.96 | 1.00 |
|  | BMI ≥ 24.0 | 9 | 14 144 | 63.6 |  | 1.95 (0.73-5.23) |  | 24 | 63 885 | 37.6 |  | 1.01 (0.62-1.65) |
|  | 21-25 |  |  |  |  |  |  |  |  |  |  |  |
|  | BMI < 24.0 | 72 | 161 967 | 44.4 | 0.007 | 1.00 |  | 200 | 354 018 | 56.4 | 0.99 | 1.00 |
|  | BMI ≥ 24.0 | 58 | 81 732 | 70.9 |  | 1.60 (1.13-2.26) |  | 111 | 196 269 | 56.5 |  | 1.01 (0.79-1.26) |
|  | ≥ 26 |  |  |  |  |  |  |  |  |  |  |  |
|  | BMI < 24.0 | 44 | 75 470 | 58.3 | 0.21 | 1.00 |  | 21 | 40 211 | 52.2 | 0.18 | 1.00 |
|  | BMI ≥ 24.0 | 27 | 34 211 | 78.9 |  | 1.35 (0.84-2.19) |  | 19 | 23 796 | 79.8 |  | 1.53 (0.82-2.84) |
